# Supplementary material for: In Silico Screening of Novel TMPRSS2 Inhibitors for Treatment of COVID-19
Source: Molecules. 2022 Jun 30;27(13):4210. doi: 10.3390/molecules27134210 (PMC9268035; doi:10.3390/molecules27134210)
Supplement: Supplementary file 1 [file molecules-27-04210-s001.zip › molecules-1728984-supplementary.pdf]

molecules

Supporting information

Table S1. The properties, energy score and hydrogen bond interactions with TMPRSS2 residues of potential TMPRSS2 inhibitors.

Chemical name	Structure	Energy* (kcal/mol)	hydrogen bond residues	Chemical Formula	Summary
Camostat	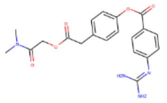	-6.2	Ser441, Ser436	C20H22N4O5	Camostat is a serine protease indicated in Japan to treat chronic pancreatitis.
Nafamostst	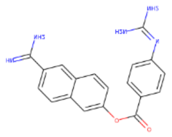	-6.1	His296, Asp435, Ser441, Gly464	C19H17N5O2	A synthetic serine protease inhibitor, Nafamostat is approved as an anticoagulant therapy in patients undergoing continuous renal replacement therapy for acute kidney injury.
ansamycin r-116	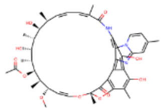	-9.7	Cys390, Gly439, Ser441, Gly464	C43H51N3O11	none
Ergotamine	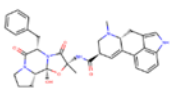	-8.9	His296, Ser441, Gly462	C33H35N5O5	Ergotamine is an alpha-1 selective adrenergic agonist vasoconstrictor used to treat migraines with or without aura and cluster headaches
Lumacaftor	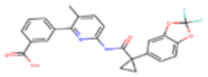	-8.9	Lys342, Ser441, Gly462, Ser463	C24H18F2N2O5	Lumacaftor is a protein chaperone used in combination with ivacaftor for the treatment of cystic fibrosis in patients who are homozygous for the F508del mutation in the CFTR gene.
Indacaterol	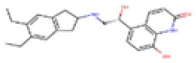	-8.8	Ser436, Cys437, Gly439, Ser441	C24H28N2O3	Indacaterol is an inhaled long-acting beta-2 adrenergic agonist used to relax bronchial smooth muscle and improve symptoms and airflow obstruction caused by Chronic Obstructive Pulmonary Disease (COPD) and moderate to severe asthma
Paliperidone	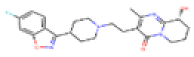	-8.7	Ser441, Gly462	C23H27FN4O3	Paliperidone is an atypical antipsychotic used in the treatment of schizophrenia and other schizoaffective or delusional disorders.
Dihydroergotamine	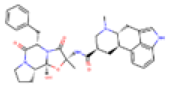	-8.7	Gly462, Ser463, Gly464	C33H37N5O5	Dihydroergotamine is an ergot alkaloid used in the acute treatment of migraine headache and cluster headache.

Raltegravir	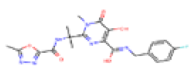	-8.7	Ser436, Ser441, Trp461, Gly462	C20H21FN6O5	Raltegravir is an antiretroviral agent used for the treatment of HIV infections in conjunction with other antiretrovirals.
Lomitapide	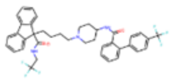	-8.7	Gly439, Gly462, Ser463, Ser441	C39H37F6N3O2	Lomitapide is a microsomal triglyceride transfer protein inhibitor used to lower cholesterol associated with homozygous familial hypercholesterolemia (HoFH), reducing risk of cardiovascular events such as myocardial infarction and stroke.
Zelboraf	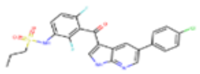	-8.7	Ser436, Cys437, Ser441	C23H18ClF2N3O3S	Vemurafenib is a kinase inhibitor used to treat patients with Erdheim-Chester Disease who have the BRAF V600 mutation, and melanoma in patients who have the BRAF V600E mutation.
Glecaprevir	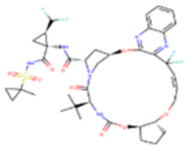	-8.6	Lys342, Gly462	C38H46F4N6O9S	Glecaprevir is a Hepatitis C NS3/4A protease inhibitor used to treat Hepatitis C.

* Affinity binding energy of virtual screening results by AutoDock vina program.

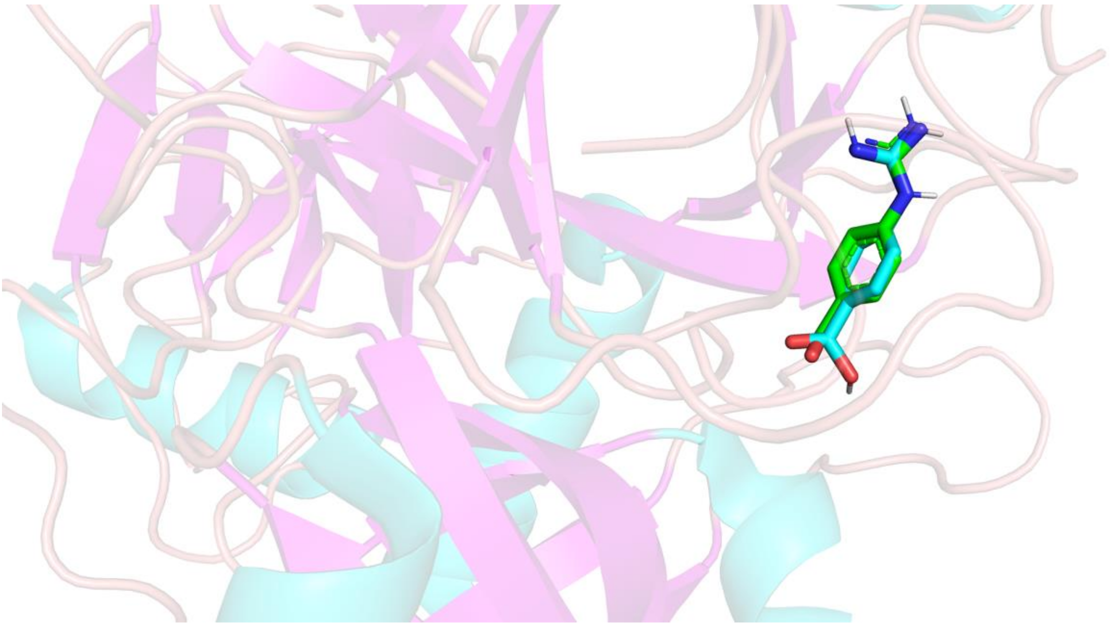

Figure S1. Superposition of Nafamostat between conformation with lowest binding free energy and crystal structure. The green stick is Nafamostat in the crystal structure, and the blue stick is the structure of Nafamostat after docking. RMSD between them is 0.508 Å.

A. Ansamycin r-116

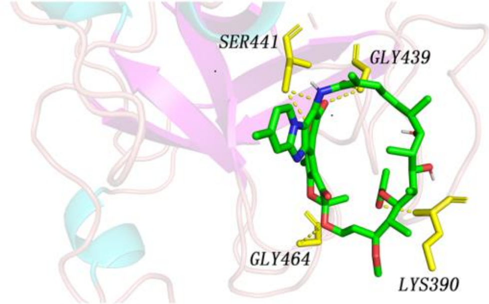

B. Ergotamine

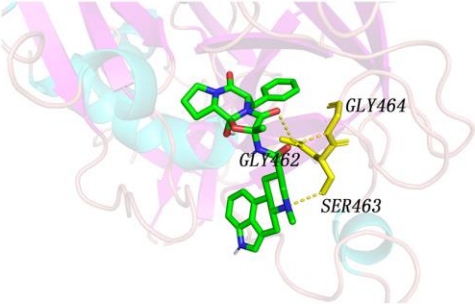

C. Lumacaftor

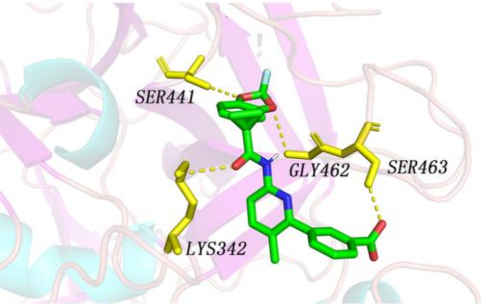

D. Indacaterol

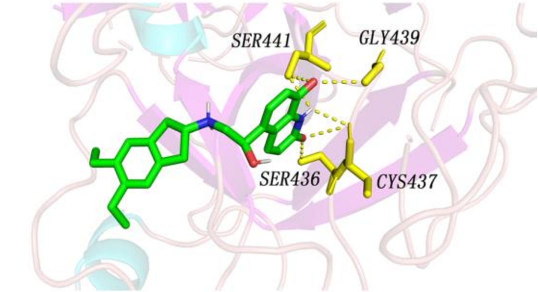

E. Paliperidone

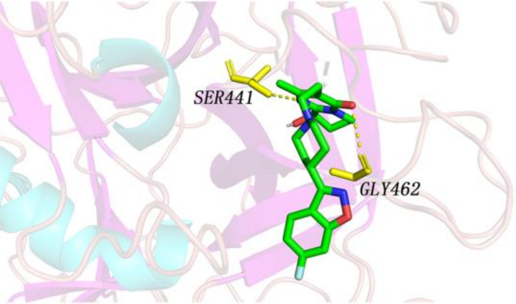

F. Dihydroergotamine

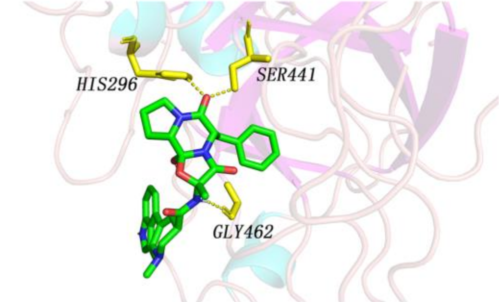

G. Raltegravir

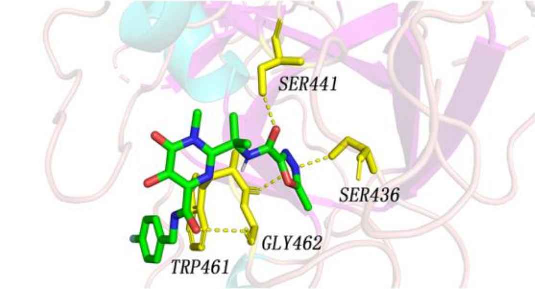

H. Lomitapide

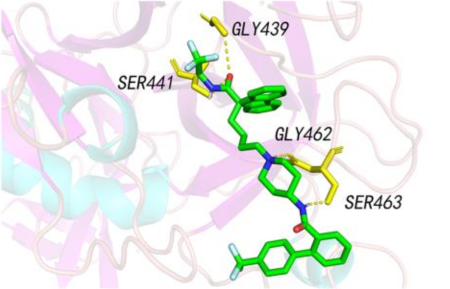

I. Zelboraf

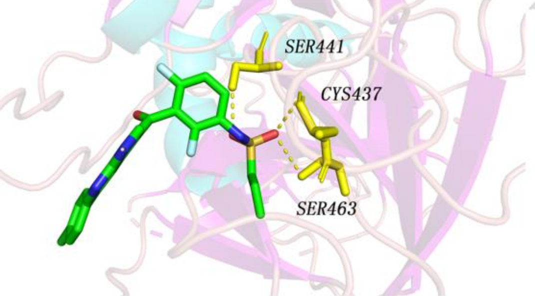

J. Glecaprevir

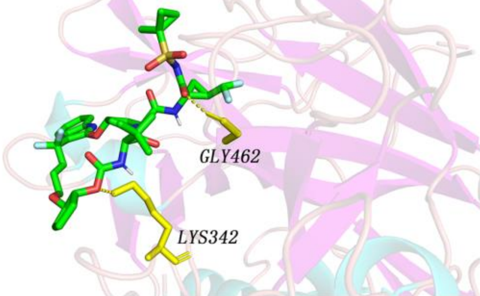

Figure S2. Interaction diagram of top10 compounds with TMPRSS2 active site. The compounds (A) ansamycin r-116, (B) Lumacaftor, (C) Ergotamine, (D) Indacaterol, (E) Paliperidone, (F) Dihydroergot, (G) Raltegravir, (H) Lomitapide, (I) Zelboraf, (J) Glecaprevir are represented as green sticks. TMPRSS2 is represented as cartoon. Amino acid residues that interact with inhibitors are shown as yellow sticks. And hydrogen bonds are represented as dashed yellow lines.

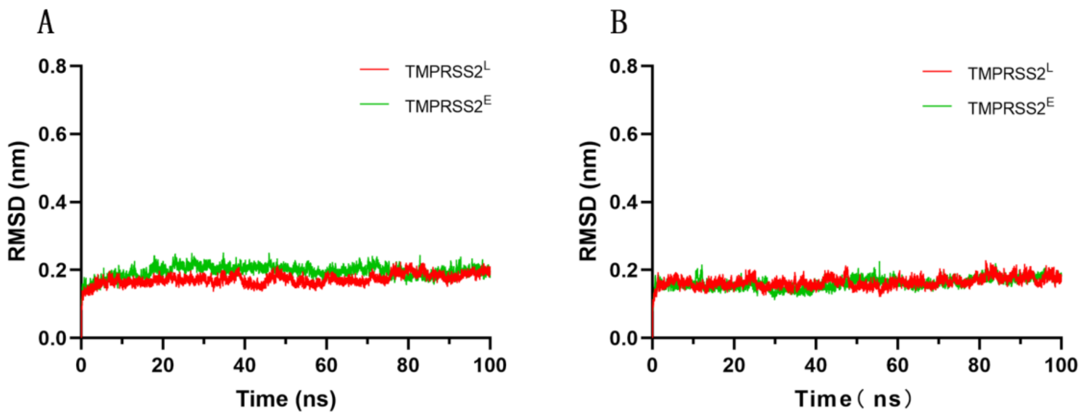

Figure S3. TMPRSS2E/L represents the protein structure of TMPRSS2 and Ergotamine/Lumacaftor complex. RMSD of proteins in the simulated data were repeated, first time (A), second time (B).

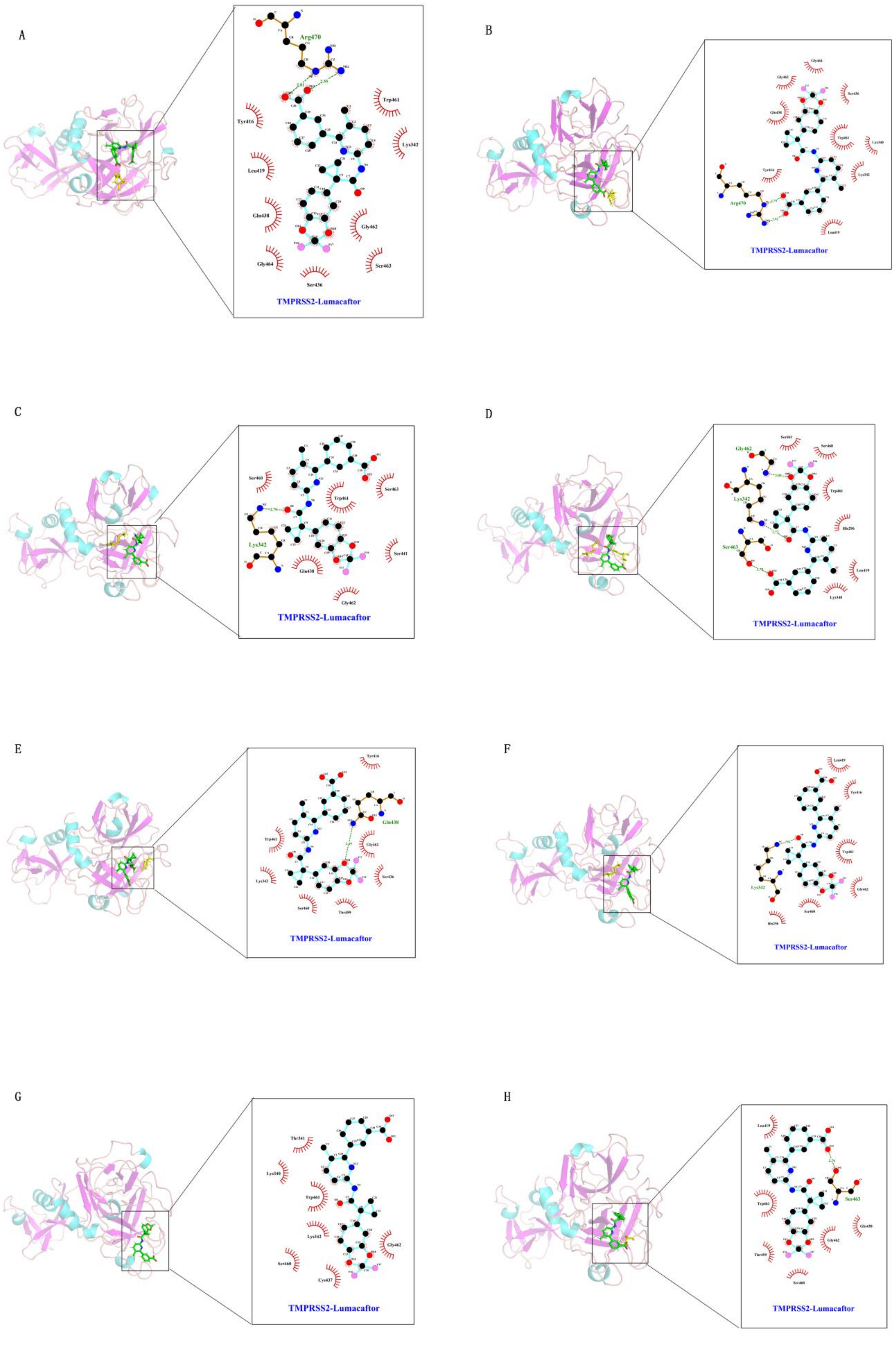
A
Arg470
Trp461
Tyr416
Lys342
Leu419
Gln438
Gly462
Gly464
Ser463
Ser436
TMPRSS2-Lumacaftor
B
Arg470
TMPRSS2-Lumacaftor
C
Ser460
Ser463
Trp461
Lys342
Ser441
Gln438
Gly462
TMPRSS2-Lumacaftor
D
Gly462
Lys342
Ser463
TMPRSS2-Lumacaftor
E
Gln438
TMPRSS2-Lumacaftor
F
Lys342
TMPRSS2-Lumacaftor
G
TMPRSS2-Lumacaftor
H
Ser463
TMPRSS2-Lumacaftor

Figure S4. The interaction between TMPRSS2, the most representative structure of the 8 clusters, and Lumacaftor. (A) first (B) second (C) third (D) fourth (E) fifth (F) sixth (G) seventh (H) eighth. TMPRSS2 is represented as cartoon. The Lumacaftor is shown as a green stick. Amino acid residues hydrogen-bonded to inhibitors are shown as yellow sticks. Dotted green lines represent hydrogen bonds, and numbers indicate the distance between donor and acceptor atoms. The red circles represent hydrophobic interactions.

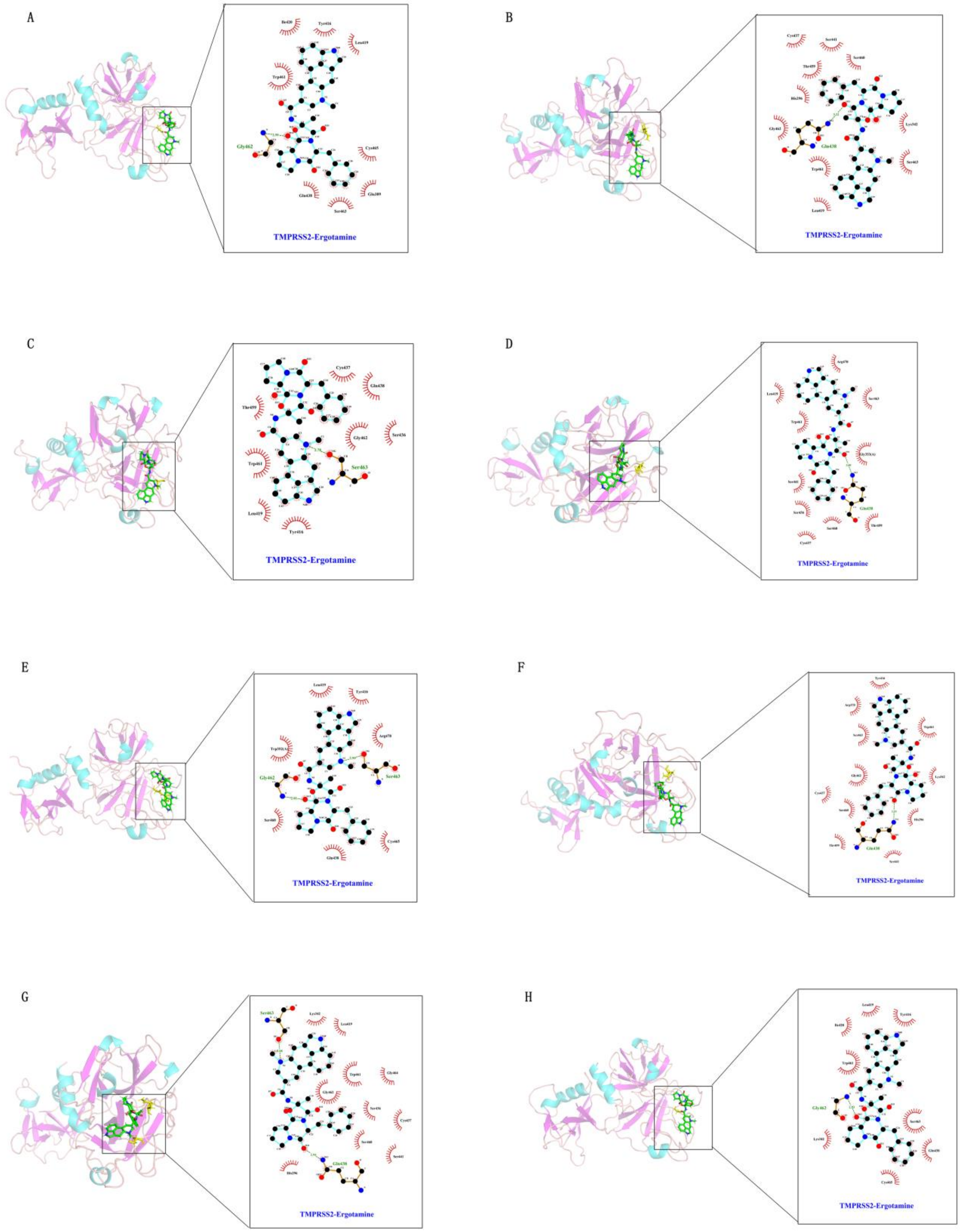

Figure S5. The interaction between TMPRSS2, the most representative structure of the 8 clusters, and Ergotamine. (A) first (B) second (C) third (D) fourth (E) fifth (F) sixth (G) seventh (H) eighth. TMPRSS2 is represented as cartoon. The Ergotamine is shown as a green stick. Amino acid residues hydrogen-bonded to inhibitors are shown as yellow sticks. Dotted green lines represent hydrogen bonds, and numbers indicate the distance between donor and acceptor atoms. The red circles represent hydrophobic interactions.
